# Oncogenic Viruses in Organ Transplantation: Implications of Virus-Host Interactions for Cancer Development

**DOI:** 10.3390/v17101299

**Published:** 2025-09-25

**Authors:** Seyed-Mahmood Seyed-Khorami, Arezou Azadi, Ala Habibian, Monireh Hosseini, Xiaofeng Fan, Hoorieh Soleimanjahi, Mahmoud Reza Pourkarim

**Affiliations:** 1 Department of Virology, Faculty of Medical Sciences, Tarbiat Modares University, P.O. Box 14115-111, Tehran 14155, Iran; ced-mahmood@modares.ac.ir (S.-M.S.-K.); azadi_arezou@yahoo.com (A.A.); monirehhosseini1374@gmail.com (M.H.); 2 Infectious Diseases Research Center, Kashan University of Medical Science, Kashan 87159-81151, Iran; habibian-ala@kaums.ac.ir; 3 Department of Microbiology, Faculty of Medicine, Kashan University of Medical Sciences, Kashan 87159-81151, Iran; 4 Division of Gastroenterology and Hepatology, Department of Internal Medicine, Saint Louis University School of Medicine, St. Louis, MO 63104, USA; xiaofeng.fan@health.slu.edu; 5 Saint Louis University Liver Center, Saint Louis University School of Medicine, St. Louis, MO 63104, USA; 6 Laboratory for Clinical and Epidemiological Virology, Department of Microbiology, Immunology and Transplantation, Rega Institute for Medical Research, KU Leuven, 3000 Leuven, Belgium

**Keywords:** transplant, oncogenes, dynamics, viral recurrence, GVHD, graft rejection, host, interaction, cancer

## Abstract

Organ transplantation significantly enhances the survival and quality of life for recipients. However, multiple dependent and independent variables can adversely affect life expectancy after transplantation. Cancer is one of the most common causes of morbidity and mortality for long-term organ transplant recipients. The incidence of cancer in transplanted tissues can be twice as high in approximately 32 distinct cancer types. Oncogenic viruses present in graft tissues may contribute to the etiology of various cancers in transplant recipients. Such oncogenic viruses include hepatitis viruses, papillomaviruses, Epstein–Barr virus, Kaposi’s sarcoma, Merkel cell virus, JC virus, BK virus, and human T-lymphotropic virus type 1, all of which have been associated with various malignancies in these patients. To mitigate this risk, a comprehensive viral screening protocol should be integrated into the transplantation process. Depending on the type of graft, diagnostic methods, control strategies, and post-transplantation care may vary considerably. To efficiently implement any strategy to inhibit viral oncogenicity, a comprehensive understanding of viral–host interactions involving oncogenic viruses within graft tissue is essential. The current view of tumor biology is that changes in the tumor microenvironment and immune signaling influence evolutionary selection pressures. Such interactions ultimately promote conditions that favor uncontrolled host–cell proliferation and malignant transformation. This review examines these viral–host interactions and their role in cancer development among transplant recipients.

## 1. Introduction

Organ transplantation has become increasingly important in healthcare. As people age, the risk of organ failure rises, making organ transplantation a vital treatment option. This medical procedure offers hope and improves the quality of life for individuals experiencing organ failure. However, organ transplantation also presents significant challenges and imposes substantial burdens. The financial demands, including the cost of surgery, immunosuppressive medications, and post-transplant care, can tremendously strain healthcare resources [1]. According to Statista.com, an estimated 157,000 organ transplants were performed globally from living and deceased donors in 2022. In 2024, the global transplantation market was valued at USD 17.3 billion, and based on a recent study by MarketDigits it is expected to exceed USD 30 billion by 2030. Nevertheless, in recent decades, organ transplantation has demonstrated improved effectiveness and survival rates [2,3].

Advancements in medical science hold great promise for the future of organ transplantation. Researchers and healthcare providers are working to improve procedures to achieve better outcomes, reduce rejection rates, and ensure long-term graft survival [4]. However, organ transplantation still faces several challenges including graft incompatibility, post-transplant infections, and cancer development, as well as ethical, and religious concerns in various communities [2].

Among post-transplant complications, infection represents a major threat. Immunosuppressive drugs, essential for preventing organ rejection, increase the body’s susceptibility to infections caused by bacteria, fungi, and viruses [5]. Viruses are of particular concern due to the potential to cause primary infections or reactivation of latent infections in the transplanted recipient. Members of *Herpesviridae* family, blood-borne viruses, and latent viruses are common examples of viruses that can cause post-transplant infection [6].

In addition, the development of cancer following transplantation is a major concern. There is a fundamental hypothesis suggesting the potential transmission of cancer through organ transplantation from donors with preexisting cancer cells. Another hypothesis proposes, the disturbances in the host immune system may contribute to tumor formation. Currently, there are no firm answers to these questions. The documented incidence of cancer transmission following transplantation is currently low, with most information being derived from sporadic case reports [7,8].

However, in both complications of post-transplantation, infection and cancer, the interaction between host and pathogen has not significantly been understood. The ability of viruses, particularly DNA viruses, to establish latent infections may offer them an evolutionary advantage by promoting cell survival and, in some cases, increasing the chance of cancer development. In such a context, oncoviruses act as agents of biological entropy, driving genetic variation and enhancing phenotypic plasticity within infected cells. The mechanisms of tumorigenesis caused by viruses in these patients remain a critical question. While a considerable number of studies have been conducted on viral infection in transplantation, further research is needed to shed light on the relationship between oncogenic viral infections and cancer development in these patients.

The host immune defense against oncogenic viruses relies critically on cytotoxic T lymphocytes (CTLs) and natural killer (NK) cells to eliminate virus-infected cells via MHC-restricted antigen recognition and perforin/granzyme-mediated apoptosis. Immunosuppressive therapies impact all arms of innate and adaptive immunity predisposed to viral infections [9,10].

The long-term interplay between oncogenic viruses and the host immune system in transplant recipients fosters permissive microenvironment for tumorigenesis through multiple synergistic mechanisms [11,12].

For example, Immunosuppressive regimens, particularly calcineurin inhibitors (tacrolimus, cyclosporine) and antimetabolites (mycophenolate mofetil), disrupt this surveillance by inhibiting T-cell activation (via NFAT blockade) and lymphocyte proliferation, and by dampening cytotoxic T-cell activity (T-cell exhaustion, by upregulated PD-1/CTLA-4 expression), allowing viral persistence, and permitting unchecked viral replication and viral oncoprotein expression [13,14].

Oncogenic viruses can be acquired during or after the transplantation procedure or can be reactivated following transplantation. Understanding the evolutionary mechanisms of these viruses sheds light on the processes behind their carcinogenic properties, which in turn facilitates the development of more effective strategies for prevention and treatment [13,15,16].

Accordingly, oncogenic viruses who have significant impact in post-transplantation complications include Epstein–Barr virus (EBV), Human herpesvirus 8 (HHV-8/KSHV), Human papillomavirus (HPV), Hepatitis B virus (HBV), Hepatitis C virus (HCV), Merkel cell polyomavirus (Merkel), BK virus, JC virus, Human Polyomavirus (HpyV), and Human T-Lymphotropic Virus-1 (HTLV-1) (Table 1) [17,18].

**Figure 1:**
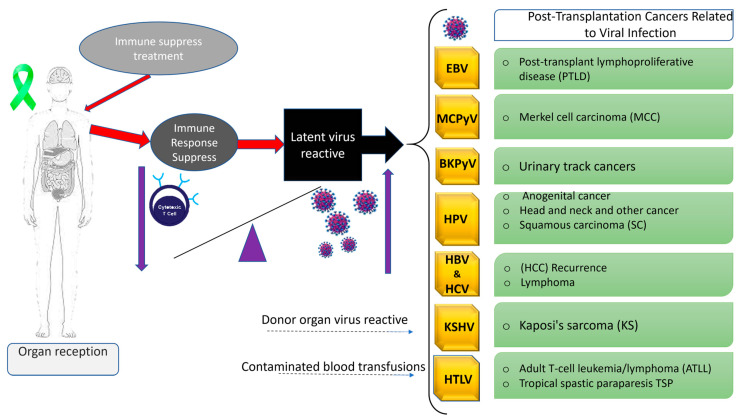
Post-transplantation cancers in transplant recipients caused by oncogenic viruses. Immunosuppressive therapy can disrupt immune balance after organ transplantation, impairing cytotoxic T-cell function. This immune suppression can trigger the reactivation of latent viruses, thereby increasing the risk of virus-associated malignancies in transplant recipients.

In this review, we aim to provide a clear picture of well-known oncogenic viruses, such as EBV, KSHV, HPV, HBV, HCV, Merkel cell virus, BK virus, JC virus, HPyV, and HTLV-1, and discuss the clinical presentation, diagnosis, treatment, and prevention strategies for these viral, highlighting their key roles in the development of virus-associated cancers in transplant recipients (Figure 1).

## 2. Oncogenic Viruses

### 2.1. EBV in Transplantation

#### 2.1.1. EBV Infection and Post-Transplant Lymphoproliferative Disease

EBV is an enveloped virus belonging to the gamma-herpesviruses. Globally, EBV infects almost 90% of the adult population [20]. Transmission of this virus occurs mainly through the exchange of saliva, and it selectively targets B lymphocytes by binding to the CD21 receptor in the throat lymphoid tissues [21]. Initial infection occurs usually in childhood before the age of 10 and is often asymptomatic [22]. However, EBV infection in adolescents is considered an etiology of infectious mononucleosis (IM), which is characterized by fever, pharyngitis, and lymphadenopathy [23]. EBV remains in a latent state for the lifetime of humans, in which viral gene expression is limited [21,24]. Establishing a chronic latent infection is a critical virus–host interaction and plays a pivotal role in the EBV life cycle [25]. EBV persists passively in the reticuloendothelial system and peripheral blood, infecting naïve B-cells, post-germinal center cells, and memory B-cells [26]. Approximately 1 in 10^2^ to 1 in 10^3^ B cells in peripheral blood cells are infected with EBV during acute infectious mononucleosis and express the latency III pattern of transcription [27]. Lytic replication may also occur in a subset of B cells in the peripheral blood cells [28]. Efficient humoral and cellular immune responses eliminate the majority of these cells, leaving a population of about 1 in 10^10^ circulating infected B cells that express different latency patterns with more restricted EBV gene expression [29]. EBV evades the immune system cells, and due to minimal gene expression, the infected cells become weak targets for EBV-specific cytotoxic T-lymphocytes [30]. Effective cell-mediated immune responses are crucial for controlling EBV infection [21]. When the host lacks adequate T-lymphocyte immunity, the virus can cause uncontrolled B-cell proliferation leading to EBV-related lymphomas [31]. EBV encodes latent proteins (e.g., LMP-1, EBNA-2) that dysregulate NF-κB and JAK/STAT signaling, manipulate cellular signaling pathways which can promote B-cell proliferation while evading immune surveillance [31].

Post-transplant lymphoproliferative disease (PTLD) is a serious complication of organ transplantation [32]. Reported survival rates for PTLD vary from 40% to 60% at 5 years after diagnosis EBV-positive PTLD often occurs within the first year post-transplantation, usually within the first 6 months whereas EBV-negative PTLD typically occurs more than 5 years after transplantation [33,34]. 

**Figure 2:**
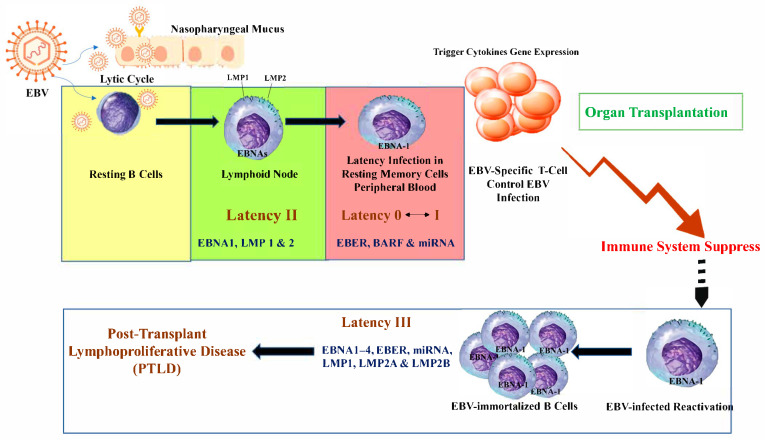
Latent EBV in transplant recipients causes PTLD through long-term persistence. EBV can persist in B cells in different latency states. Under immunosuppression, such as after organ transplantation, EBV can reactivate, leading to uncontrolled B-cell proliferation and the development of PTLD.

Active cell-mediated immune responses are an essential part in controlling EBV infection [21]. Dynamically, there is a balance between viral load and immune surveillance that maintains persistent infection at a subclinical level. However, the administration of immunosuppressive agents during organ transplantation procedures can disrupt this balance and eventually lead to the reactivation of EBV. The loss of CTL activity likely allows infected cells to express a latency III pattern. leading to the accumulation of virus-infected B cells and amplification of virus replication (Figure 2) [35,36]. In immunosuppressed patients, the number of EBV-infected memory B cells can be as much as 50 times higher than that in a healthy carrier [37]. Therefore, following transplantation, there is an increase in the level of detectable EBV DNA in peripheral blood cells as well as EBV shed in saliva. These changes return to normal dynamics when the level of immunosuppression is reduced [37,38].

PTLD does not develop in every EBV-seropositive transplant recipient. Most infected lymphoblast transit into resting memory cells, and only B cells that are unable to make this transition will begin to proliferate overtly. In the presence of a high level of viremia, cells other than naive B cells, such as germinal center B cells and memory B cells, may also be infected incidentally. These so-called bystander B cells may express a latency III phenotype, but are unable to switch to latency II, and uncontrolled growth of these bystander cells could lead to PTLD [39]. Both EBV primary infection and reactivation are risk factors for the development of PTLD. EBV-naive patients have a much higher risk of developing PTLD than latently infected patients who are EBV-seropositive at the time of organ transplantation [40]. This is more likely to occur when the recipient is serologically EBV-negative, and the donor is serologically EBV-positive [41].

#### 2.1.2. EBV-Associated PTLD: Incidence and Risk Factors

PTLD is a relatively common malignant complication after organ transplantation. Development of EBV-PTLD is influenced by numerous risk factors, including the pretransplant EBV-serostatus, the type of organ transplanted, and the age of the individual organ recipient [42].

Current data indicate that the most common types of transplants were kidney (41%) and liver (31%). PTLD is seen in up to 10–15% of all SOT adult recipients. Small intestine transplant recipients are at the highest risk for development of PTLD (up to 32%), while recipients of pancreas, lung (5.7 per 1000 person-years), heart (2.2 per 1000 person-years), and liver (2.4 per 1000 person-years) transplants are at moderate risk (3–12%). Renal (1.6 per 1000 person-years) transplant recipients are at relatively low risk (1–2%) [43].

A higher frequency of PTLD after transplantation has been observed in children, particularly among pre-transplant EBV-seronegative individuals, with rates ranging from 4–22%. In contrast, the incidence among adult organ transplant recipients is relatively low, about 1 to 2% [44].

#### 2.1.3. EBV-Associated PTLD: Diagnosis

The disease burden at the time of diagnosis closely correlates with prognosis. Therefore, early diagnosis is crucial to maximize the chances of a successful treatment and/or prevention of PTLD [42]. The diagnosis is based on histological and clinical backgrounds [32]. Since primary EBV infection is an important risk factor for developing PTLD, the EBV-serostatus of the donor and the recipient is determined before organ transplantation. EBV viral capsid antigen (VCA), EBNA-1, and early antigen antibodies are detected through serological assays [43]. After immunosuppressive therapy, serological tests have limited or no value in post-transplantation settings due to absent or delayed humoral immune responses. Therefore, serology tests should not be considered as a diagnostic tool for PTLD [44]. A significant correlation between the increase in viral load and the development of EBV-related PTLD has been reported. Quantitative PCR may be useful for diagnosis and monitoring of PTLD, although the EBV viral load level over time in the same patient may show substantial variability [45]. Monitoring of EBV viral load in lymphocytes and whole blood cells is more sensitive and less specific compared with plasma monitoring. EBV plasma monitoring shows greater specificity for PTLD detection [46,47].

A promising adjunctive laboratory test to improve specificity is the measurement of the EBV-specific T-cell response. This response is decreased in the immunosuppressed organ transplant recipients. A combination of a high viral load and a low EBV-specific T-cell response seems to have a strong positive predictive value for the progression of PTLD [48].

#### 2.1.4. EBV-Associated PTLD: Treatment and Preemptive Intervention Strategy

Closely monitoring the EBV viral load in high-risk patients, such as EBV-seronegative organ transplant recipients of EBV-positive organ donors, can be helpful for preemptive intervention [49]. Post-transplantation monitoring of donors is advised to occur twice weekly, but this may present logistical challenges [50]. After diagnosis, PTLD is treated based on different strategies, including reduction in immunosuppression to modulate immune function, administration of immunoglobulins, and tumor resection if it is feasible [51]. Generally, the proliferated cells in PTLD are predominantly latently infected. Antivirals such as acyclovir and ganciclovir can inhibit lytic DNA replication, which, by reducing lytic replication, decreases the infection of B cells and latently infected cells, eventually, reducing the risk of PTLD [52]. The administration of EBV-specific CTLs is another approach to boost the host’s EBV-immune response [51].

#### 2.1.5. EBV-Associated PTLD: Prognosis

According to time-bound events, PTLD is categorized into two levels. Indeed, in the first year after transplantation, there is an early onset of PTLD, while the late onset occurs thereafter. It has been demonstrated that EBV is positive in the early stages of PTLD; furthermore, EBV remains detectable 4–5 years after transplantation which depicts a bi-modal pattern of PTLD. Nevertheless, the precise role of EBV in the prognosis of PTLD remains poorly defined [53].

### 2.2. KSHV in Transplantation

Human herpesvirus 8 (HHV-8), also known as Kaposi’s sarcoma-associated herpesvirus (KSHV) is a member of the *Herpesviridae* family that has been directly associated with Kaposi’s sarcoma (KS) malignancy [52]. KSHV, has been recognized as a crucial factor in the development of Kaposi’s sarcoma, as evidenced by the finding of HHV-8 DNA in almost all KS patients [53]. In transplant recipients, a strong association between KSHV and KS has been confirmed [54]. KSHV inflammatory cytokine syndrome (KICS) is characterized by high viral loads and elevated interleukin-6 secretion in infected patients [55].

Based on geographic areas, seroprevalence of HHV-8 varies. Remarkably, higher-than-average seroprevalence has been reported in Sub-Saharan Africa, the Mediterranean region, and the Xinjiang region of China, which are considered endemic areas. In contrast, the United States and Western Europe have a low seroprevalence rate in the general population. [56]. KSHV can be transmitted through sexual contact, blood transfusion, tissue transplants, and saliva [57]. Like other γ-herpesviruses, the KSHV life cycle is divided into two distinct phases: the lytic phase and the latent phase, which are recognized by different viral gene expression patterns [58]. KSHV establishes lifelong latency within the host, with sporadic reactivation and lytic replication. During latency, the KSHV genome exists as an extra-chromosomal viral genome (episome) with minimal expression of a subset of latent viral proteins: K1, K12/Kaposin, K13/vFLIP, ORF73/LANA, ORF72/vCyclin and vIL-6 [59]. KSHV is known to infect endothelial and lymphoid cells; however, its viral tropism is broader than initially thought [53]. For instance, using in situ hybridization, the virus has been detected in kidney tubular epithelial cells and glomerulo-endothelial cells, as well as in cases of acute primary KSHV infection affecting newborns [60].

#### 2.2.1. KSHV-Related Cancers: Incidence and Risk Factors

Kaposi sarcoma is clinically characterized by vascular lesions and often presents as violet to purple plaques on the skin or mucocutaneous surfaces. These types of lesions may be painful and sometimes accompanied by lymphedema, or secondary infections [61]. Kaposi sarcoma has a tropism for the gastrointestinal system and lungs, with lung involvement being associated with mortality due to KSHV [62,63,64].

KSHV deploys viral FLICE inhibitory protein (vFLIP) K13 and cyclin homologs (vCyclin, a homolog of host Cyclin D) to inhibit apoptosis and promote cell-cycle progression [65].

Due to inflammation and immune suppression, infected cells can persist and proliferate. KSHV encodes the latency-associated nuclear antigen (LANA), which binds to p53, suppresses apoptosis, and prevents Fas-induced programmed cell death [63]. In addition, KSHV induces c-kit expression and alters the original cuboidal monolayer into the spindled morphology characteristic of Kaposi sarcoma cells [21]. KSHV also upregulates matrix metalloproteinases which are attributed to tissue remodeling and tumor progression [63].

The close virus–host interaction of KSHV occurs during the latency phase. This is followed by intermittent reactivation of the virus from latency, enabling lytic replication, production of infectious progeny, and overexpression of lytic viral genes during the lytic cycle. This promotes viral adaptation by producing genetic diversity, particularly through emerging mutations in the IR1 region and in latency-associated nuclear antigen (LANA), which may accumulate and evolve over time [57,58,59]. The frequency of Kaposi sarcoma associated with KSHV in post-organ transplantation is 500–1000 times higher than in the general population. This incidence is associated with immunosuppressive therapy. The reported incidence of post-transplant KS ranges from 0.5 to 5% and differs depending on the patient’s country of origin and the type of organ received [66]. It is not completely clear whether post-transplantation KS is due to the KSHV reactivation as a result of immunosuppression or to primary KSHV infection transmitted via the donor organ transplantation [67,68]. In the case of kidney transplantation, the analysis of the sequence of the KSHV ORF-K1 membrane protein gene and the ORF-73 gene demonstrates that the isolated strains from the KS lesions of the recipient kidney had originated from the organ donor [69,70]. However, reactivation appears to play a greater role in the risk of KS than do new acute infections [66]. The rapid onset of tumor formation (average, 7 months) in KS patients just after the beginning of immunosuppression shows the critical role of immune surveillance in preventing KS among KSHV-infected individuals. Retrospective studies reported that 23–28% of KSHV—seropositive recipients developed KS, and there was no difference in terms of graft loss and survival between KSHV seronegative and seropositive patients [71].

#### 2.2.2. KSHV-Related Cancers: Diagnosis

Several studies have unraveled the potential use of screening for KSHV antibodies targeting latent nuclear antigens among organ donors/recipients. However, screening for KSHV, even in geographical regions with low KSHV infection prevalence, remains controversial [66]. The lack of a standardized protocol for serological assays to detect anti-KSHV antibodies is the major limitation for the universal implementation of screening programs for organ donors/recipients in transplantation centers [72]. Previous studies have demonstrated that the seroprevalence of KSHV antibodies against latent antigens in low-risk populations is very low. In this study, whole cells treated with phorbol esters in an IFA assay were used to induce lytic phase viral antigens, which were found to have higher rates of seropositivity in various low-risk populations [71].

#### 2.2.3. KSHV-Related Cancers: Prevention and Treatment

Because KSHV is transmitted through saliva, blood, and sexual contact, transplant candidates should avoid high-exposure behaviors, e.g., deep kissing, unprotected sex. Depending on disease extent, immune status, and clinical presentation, current treatment approaches for Kaposi’s Sarcoma (KS) include Antiretroviral Therapy (ART) for HIV-associated KS, local therapies such as surgery, radiation, or chemotherapy for limited lesions, and systemic therapies like Liposomal Doxorubicin for advanced cases [73,74].

#### 2.2.4. KSHV-Related Cancers: Prognosis

In solid organ transplant recipients, Primary Effusion Lymphoma (PEL), a rare type of non-Hodgkin lymphoma, typically develops within the median timeframe of 8 years post-transplantation. Of note, the prognosis of PEL is very poor [75,76]. Furthermore, post-transplant KS (PT-KS) typically develops within the first year after transplantation along with skin lesions. Moreover, visceral involvement occurs in up to 50% of liver transplant recipients and is associated with a high mortality rate. PT-KS may occur simultaneously with MCD in solid organ transplant recipients [77].

### 2.3. HPV in Transplantation

Human papillomavirus (HPV) is a small double-stranded DNA virus (family *Papillomaviridae*) that infects mucous membranes and basal epithelial cells of the skin [78]. HPV is transmitted via Close direct person-to-person contact [79]. Many early infections of the cervix clear or become undetectable within 2 years after initial detection [79]. There are over 200 different HPV subtypes, classified as high-risk and low-risk types based on their propensity to cause invasive cancer. Low-risk types are accompanied by anogenital warts, mild cervical dysplasia, and recurrent respiratory papillomatosis. On the other hand, 12 high-risk genotypes, mainly including types 16 and 18, are the most common HPV types found in cancers [80,81]. Persistent infection with high-risk HPV types such as 16 and 18 can disturb the normal replication of epithelial cells and increase the risk of malignancy transformation [82].

Cell-mediated immunity (CMI) plays a critical role in controlling HPV infections. Immunosuppression in organ transplantation impairs the ability to clear new HPV infections and can reactivate latent HPV infections. Transplant recipients have a significantly increased risk of HPV-associated malignancy compared with the general population [83]. There are no reports of HPV being acquired through transplantation [84].

#### 2.3.1. HPV-Related Cancers: Incidence and Risk Factors

Oncogenic DNA viruses such as HPV and EBV contribute to tumor development through genetic instability, epigenetic variations, and immune evasion.

HPVs express E6 and E7 oncoproteins that degrade p53 and retinoblastoma protein, disabling cell-cycle checkpoints [85,86].

HPV infection is associated with 90% of cervical and anal cancers and also, as well as other related cancers in the general population [87]. Cervical carcinoma is a leading cancer and a major cause of death among women globally [88]. For organ transplant recipients, immunosuppressive medications hinder the body’s ability to fight new HPV infections, thereby increasing the risk of HPV-related cancers [89]. Kidney transplant recipients exhibit a substantially increased long-term cancer risk of HPV-related cancers which plays a crucial role in cancer progression and an earlier average age of onset. Kidney transplant recipients face drastically elevated risks of HPV-associated cancers including up to 100-fold increased risk of anal cancer, a 50-fold elevated risk of vulvar cancer, and a 14-fold more risk of cervical cancer [90].

Nonmelanoma skin cancer occurs 65–250 times more frequently in transplant recipients than in the general population and is often characterized by an earlier age of onset [91]. There is less evidence for a direct role between squamous carcinoma (SCC) of the skin and HPV infection [89,90]. Oropharyngeal neoplasia is a subset of head and neck cancer, a type of SCC that develops in the oropharynx, including the tonsils, base of tongue, soft palate, and posterior pharyngeal wall. A significant subset is linked to high-risk HPV (especially HPV-16). Transplant recipients have a higher risk of HPV-related oropharyngeal cancer due to chronic immunosuppression followed by HPV persistence [92,93].

HPV can also cause a benign upper-airway neoplasm called recurrent respiratory papillomatosis. However, studies are conflicting regarding the role of HPV in the pathogenesis of pulmonary neoplasms in transplant recipients, necessitating further research [94].

#### 2.3.2. HPV-Related Cancers: Diagnosis

During each visit, it is crucial to conduct a comprehensive external examination of the skin to check for the presence of cutaneous warts in transplant recipients. Note that the appearance of any anogenital wart in a transplant recipient should warrant further investigation as it may indicate the presence of HPV-related lesions. The vulva, vagina, anus, and cervix should also be carefully examined during each visit [95]. Transplant centers recommend that transplant recipients undergo the HPV-infected women screening protocol [96]. The cervical Pap smear test should be performed every 6 months during the first year after transplantation [97]. The use of high-risk HPV testing (PCR and DNA in situ hybridization) is recommended for transplanted women in conjunction with a Pap test in the general population. If both tests are negative, the screening interval can be increased from every three years to every five years. Immunocompromised women are screened every 6–12 months [91].

#### 2.3.3. HPV-Related Cancers: Prevention and Treatment

The HPV vaccines contain HPV L1 capsid proteins alone or in combination with L2 minor capsid proteins, both of which self-assemble into virus-like particles (VLP) [98]. Three prophylactic HPV vaccines are available include Cervarix, a bivalent vaccine, targeting (HPV types 16 and 18), Gardasil, a quadrivalent vaccine (HPV types 6, 11, 16, 18), and Gardasil 9 (HPV types 16, 18, 6, 11, 31, 33, 45, 52, 58) [99]. The treatment of HPV-associated disease in transplant recipients varies depending on the location, size, and grade of the lesion [100]. For cervical cancer, the choice of treatment depends on its stage [101]. Therapeutic options include cold-knife conization, cryotherapy, fertility-sparing surgery (uterus preserved), radical hysterectomy, or primary radiation therapy (with or without chemotherapy).

#### 2.3.4. HPV-Related Cancers: Prognosis

Solid organ transplant recipients are at an increased risk of developing HPV-related cancers compared to the general population, so implementing prevention strategies like vaccination is essential to reduce the incidence of these cancers in transplant patients [102].

### 2.4. Hepatitis Viruses in Transplantation

#### 2.4.1. HBV-Related Cancers in Transplantation

Hepatitis B virus is a partially double-stranded DNA virus (family *Hepadnaviridae*) that causes various liver diseases, including acute and chronic hepatitis, cirrhosis, and nearly half of all hepatocellular carcinoma (HCC) cases [94]. Factors involved in HCC development and progression include DNA damage, chronic inflammation, epigenetic changes, chromosomal instability, cellular senescence, telomerase reactivation, and early neo-angiogenesis [95]. The risk of developing HCC is associated with HBV genotype, genomic mutations, and the level of HBV replication [96].

Hepatitis B induces HCC through multiple oncogenic pathways, induction of oxidative stress, chromosomal instability (integration in the host genome), epigenetic modifications, interference with onco-suppressor pathways (role of X protein), activation of cancer-promoting pathways (e.g., Wnt/β-catenin pathway) [103,104].

Immunosuppressive therapy during the post-transplant period enhances HBV replication. The re-emergence of HBV DNA and its surface antigen (HBsAg) in the serum of a patient with pre-existing HBV infection following liver transplantation is referred to as reinfection [105]. HBV reactivation is defined as a more than 10-fold increase in HBV DNA levels in the serum of patients [106]. The median time to reinfection after liver transplantation was approximately of 145 days, with a few cases progressing to fibrosing hepatitis and death within 1 year of transplantation [107]. Extrahepatic reservoirs of HBV, predominantly the spleen and the peripheral blood mononuclear cells, are the most likely sources of HBV reinfection after organ transplantation [108]. Extrahepatic reservoirs of HBV rapidly enter graft hepatocytes, injure the liver, and elevate aminotransferase levels within several weeks [109]. Most reinfection cases occur in those who are hepatitis B e antigen (HBeAg) (HBeAg)–positive with elevated HBV DNA levels in the serum at the time of transplantation [110]. Before the introduction of effective antiviral agents for hepatitis B infection therapy, the presence of HBV infection was considered a contraindication to transplantation [111]. Hepatitis B reactivation, in liver transplantation for end-stage liver disease due to HBV, increased the incidence of graft loss in recipients within 1–2 years and was associated with a mortality rate of up to 50% in the first 3 years following organ transplantation [112].

##### HBV-Related Cancers: Prevention and Treatment

HBV vaccine programs and potent antiviral treatments have decreased the incidence of new HBV infections in many countries, but the rate of chronically infected individuals remains high worldwide [113]. The two primary approaches to preventing HBV reactivation after transplantation are preemptive therapy and prophylactic measures, in addition to routine vaccination of all non-immune recipients [102]. The long-term administration of intravenous HBIG significantly reduced the incidence of recurrent HBV, especially in HBeAg-negative and HBV-DNA-negative patients [112].

##### HBV-Related Cancers: Prognosis

There was no observed reactivation of hepatitis B virus in non-liver transplant recipients who are HBcAb(+)/HBsAg(−). It seems that prophylaxis might not be necessary for them. On the other hand, prophylactic strategies are indefinitely required in liver allograft recipients from HBcAb(+)/HBsAg(−) donors because they are at high risk of HBV reactivation [114].

#### 2.4.2. HCV-Related Cancers in Transplantation

HCV is a small, enveloped, single-stranded RNA virus belonging to the *Flaviviridae*. In 75 to 85% of HCV-infected patients, chronic infection develops and persist for at least 6 months [102]. The frequency of chronic hepatitis infection varies with age, race, sex, and immune system status [115]. Long-term infection with HCV is associated with various clinical complications, including fibrosis, cirrhosis, portal hypertension, and HCC [116]. It is estimated that up to 20% of individuals with chronic HCV infection will develop liver cirrhosis within a 20- to 25-year timeframe, significantly increasing their risk of HCC [117].

Cirrhosis and HCC due to chronic HCV are the leading indications for liver transplantation. Chronic HCV can lead to HCC by numerous interrelated mechanisms, including induction of oxidative stress-mediated Ca^2+^ homeostasis and genomic instability via core and NS5A proteins, while impairing interferon responses [118,119].

Reinfection after liver transplantation is common, and chronic liver disease develops in at least 70% of patients within three years, with an accelerated course [120]. Viral titers are low immediately post-transplantation but then increase by 48 h post-transplantation, peaking at four months. These titers can rise 10- to 100-fold, with the four-month titer believed to indicate future histological activity [121]. One of the recurrence patterns is acute hepatitis mainly with elevated transaminases. Another is the development of chronic hepatitis, which shows a more accelerated progression compared to chronic hepatitis in the non-transplant population [122]. It leads to cirrhosis in 25% of patients within five years [123]. The risk of cirrhosis in a transplant patient is between 17–42% within the first year, significantly higher than the rate in the immunocompetent population, which is 28% over ten years [124,125].

##### HCV-Related Cancers: Prevention and Treatment

One of the most effective treatment strategies for HCC is liver transplantation, which holds significant potential for positive patient outcomes [126]. For carefully selected HCC patients, liver transplantation can yield excellent long-term outcomes, with a five-year survival rate ranging from 60 to 70% [127]. However, recurrent disease, such as from hepatitis viruses, remains a persistent problem after liver transplantation with rates ranging from 9 to 16% [128,129]. A first peak of intrahepatic recurrence is typically seen within the first 24 months [121]. Several predictors of HCC recurrence are well known tumor size, vascular invasion, degree of differentiation, number of nodules, age, and liver inflammation [126,130].

It is important to monitor for HCV reinfection after a liver transplant. Direct-acting antivirals (DAAs) are considered safe for use 8–12 weeks post-transplant. The treatment of hepatocellular carcinoma includes various options such as surgery, liver transplantation, and systemic therapies.

##### HCV-Related Cancers: Prognosis

During the first year after transplantation, among heart, liver, and kidney transplant recipients, the mortality rates were equal. Notably, no patient deaths were found to be directly attributable to HCV infection, and HCV RNA was undetectable in the recipients before death [131].

### 2.5. Polyomaviridae Family (PyVs)

PyVs are icosahedral, non-enveloped viruses with a 5.5 Kbp double-stranded viral DNA genome [27], divided into five genera: *Alphapolyomavirus*, including human PyVs (HPyVs) 5, 8, 9, 12, and 13; *Betapolyomavirus*, including HPyVs 1 and 4; *Deltapolyomavirus* which includes only HPyVs 6, 7, 10, and 11 [132]. Some PyVs are associated with cancer in animal models, PyVs produce a wide range of pathological lesions, including various neoplastic tumors, and serve as viral agents of oncogenesis [133]. Various studies conducted over the last decade have identified almost all known HPyVs in human tumors [134].

#### 2.5.1. Merkel Cell Polyomavirus (MCPyV)-Related Cancers in Transplantation

Merkel cell polyomavirus (MCPyV) was discovered in 2008 and has been proposed as the etiologic agent of Merkel cell carcinomas (MCC) because MCPyV is detected in up to 80% of MCC cases [135]. MCPyV is acquired during childhood and is found in the skin of about 60–80% of healthy individuals. The viral replication activity of MCPyV might increase following conditions of immunosuppression and an increased risk of MCPyV-driven MCC [136,137].

MCPyV is integrated into the genome of MCC cells, a fact determined by whole-transcriptome sequencing. Clonal integration of MCPyV and long-term sunlight exposure, which leads to ultraviolet-mediated DNA damage, are two main etiologies of the carcinogenesis process of MCCs [135]. MCC was thought to originate from Merkel cell precursors, which are likely derived from hair follicle stem cells, epidermal or dermal fibroblasts, or pre-B/pro-B cells, although the exact cells of origin in MCC are not yet fully established at present. Although MCC is relatively infrequent compared to other forms of skin neoplasm, after melanoma, MCC is the second most frequent cause of death from skin malignancy. Certain demographic characteristics are considered, including older age, extensive exposure to ultraviolet (UV) light, immunosuppression, and Caucasian ethnicity, and are deemed significant risk factors for MCC development [138].

Patients with organ transplants are generally at a higher risk (between 66 and 182-fold) of developing skin cancers, including MCC, compared to the non-transplanted population [139]. In individuals who have received a kidney transplant, MCC tends to follow a more aggressive clinical course and is typically diagnosed at a younger age [140]. Such patients generally present with localized disease, a red or violaceous nodular lesion in sun-exposed skin, lymphovascular invasion, and metastasis [141,142]. Systemic immunosuppression shows the strongest association with poor survival prognosis and metastasis following kidney transplantation [142]. The mortality rate is nearly doubled in MCC-specific immunosuppressed patients compared with non-immunosuppressed patients over 3 years of follow-up [143].

##### MCPyV-Related Cancers: Prognosis

Among immunocompetent patients, solid-organ transplant recipients diagnosed with MCC tend to develop the disease at a younger age and experience a poor prognosis, characterized by a higher risk of disease progression and mortality [144,145].

#### 2.5.2. BK Polyomavirus (BKPyV) and Kidney Transplantation

The BKPyV was first isolated from the urine of a kidney transplant recipient. Primary infection regularly causes subclinical or mild respiratory disease, usually acquired in childhood. BKPyV seroprevalence in adults is over 80% [26,146]. After the primary infection, the virus becomes latent in the uroepithelium and renal tubular epithelial cells. Following immunosuppression, BKV reactivation initiates a cascade of events, resulting in the lysis of tubular cells and viruria [26,147]. The BKPyV then multiplies in the interstitium, triggering viremia and leading to various tubulointerstitial lesions, interstitial nephritis (BKVN), and allograft failure in kidney transplant recipients [148,149].

In 2012, the International Agency for Research on Cancer (IARC) classified BKPyV and John Cunningham Polyomavirus (JCPyV) as group 2B, indicating that they are possibly carcinogenic to humans [150]. BKV’s large T-antigen (TAg) binds and inactivates p53 and Rb, disrupting cell-cycle control [151,152].

In early studies, BKV large T antigens have been detected in bladder, prostate, and kidney tumors, but several researchers have failed to detect BKPyV DNA in the cancer samples. Notably, BKPyV infection/reactivation has been linked to urinary tract cancers in kidney transplant recipients according to many recent case reports [153,154]. Cells infected with BKPyV may have two different outcomes: (1) the host cell allows replication of the virus, progeny virion production, and cell lysis; or (2) the host prevents virus replication, and the continuous expression of the T antigen causes abortive infection or cellular transformation [155].

Tumors have a vital effect on the long-term survival of kidney transplant recipients; as a result, the correlation between BKPyV infection and tumorigenesis in immunocompromised individuals requires more attention. While the precise role of BKPyV in human cancers remains unclear, its strong association with the development of urothelial cancer following transplantation is well-established. According to research, kidney transplant recipients compared to the general population, are three times more likely to develop urothelial cancer [149]. BKPyV is prone to reactivation in immunocompromised populations, especially transplant recipients [156]. Reactivation of BKPyV after renal transplantation leads to symptoms including viruria, viremia, ureteral stricture, and BK virus nephritis (BKVN), whereas following hematopoietic stem cell transplantation, it can also cause hemorrhagic cystitis [157].

However, the effects of BKPyV infections on bladder cancer and the biological characteristics of tumor cells expressing BKV-related proteins remain unknown. Some studies have shown a 4 to 12-fold elevated risk of bladder cancer with evidence of BKV-associated decoy cells in the urine, biopsy-proven BKVN, or BK viremia in kidney transplant recipients [158,159]. Conversely, the relatively frequent detection of BKV in bladder cancers from transplant recipients is not necessarily indicative of a direct relationship, as this virus is detected only infrequently in bladder cancers that arise in the general population [160].

##### BK-Related Cancer: Diagnosis

Published data show evidence of BKPyV presence at tumor sites, as confirmed by polymerase chain reaction (PCR) tests detecting BKPyV DNA, immunohistochemical staining identifying BKPyV proteins, and whole-genome shotgun sequencing indicating chromosomally integrated BKPyV [161,162].

##### BK-Related Cancer: Prognosis

Disruption and loss of the transplant in a large proportion of BKV-associated nephropathy patients may occur approximately 79% of transplant survival within 3 years. In addition, the associated risk of developing bladder cancer in these patients may lead to re-transplantation [161,163].

#### 2.5.3. John Cunningham Polyomavirus (JCPyV)

JCV T-Ag causes DNA damage by inhibiting homologous recombination DNA repair (HRR) in the host cell, thereby resulting in genetic instability in infected cells [151,164]. A serological study has indicated that asymptomatic JCPyV infection occurs in approximately 90% of the adult population. JCPyV may be activated leading to cell lysis under immunosuppressive conditions such as HIV infection or transplantation; therefore, it is a confirmed etiologic factor of demyelinating progressive multifocal leukoencephalopathy (PML) [165,166]. Furthermore, JCPyV may cause lower urinary tract symptoms in males or chronic idiopathic intestinal pseudo-obstruction [167]. JCPyV is detected in several autoimmune brain diseases, including multiple sclerosis (MS), psoriasis, and Crohn’s disease, which theoretically could be considered as one of predisposing factors for PML [168].

In cells that do not support viral replication, JCPyV infection results in either abortive infection or malignant transformation [169]. Pathological examination and animal experiments have demonstrated that the JCPyV T antigen may induce tumorigenesis in gastroenterological systems, neural tissues, and the breast. Thus, JCPyV might be an etiological risk factor for tumorigenesis and should be considered in the tertiary prevention and treatment of cancer. JCPyV enters eukaryotic cells and integrates into genomic DNA [170].

##### JC-Related Cancer: Prognosis

Although in solid organ transplant patients JCV may lead to fatal disease, graft survival in individuals with and without JCV viruria is similar, and there is a favorable prognosis after transplantation [171].

#### 2.5.4. Trichodysplasia Spinulosa Polyomavirus (TSPyV)

Trichodysplasia spinulosa (TS) is a rare dermatological disease caused by Trichodysplasia spinulosa polyomavirus (TSPyV) in immunosuppressed patients, frequently following solid organ transplantation [172]. The seroprevalence of TSPyV in immunocompetent adults is around 65–80%, and the incidence of TS in immunosuppressed patients remains low, suggesting that TS is underdiagnosed and that additional unidentified may contribute to the development of TS [172,173].

The identification of TSPyV nucleic acids in tonsillar tissue has led to the speculation that lymphoid tissue may serve as a latency site for the virus [29]. Proposed mechanisms for trichodysplasia spinulosa in immunosuppressed individuals include primary, recurrent, and/or reactivation of latent TSPyV infection [174].

##### TSPyV Diagnosis

TSPyV has been identified in histopathological examination of skin biopsies, blood, tonsils, urine, cerebrospinal fluid, and respiratory specimens using various laboratory procedures, including nucleic acid detection, immunohistochemistry, and electron microscopy [175,176]; hence serologic tests are not necessary to establish the diagnosis of trichodysplasia spinulosa [174].

### 2.6. Human T-Lymphotropic Virus-1 (HTLV-1): Cancer and Incidence

Human T-lymphotropic virus type 1 (HTLV-1) is a member of *Retroviridae* family with single-stranded RNA. Despite infecting approximately 10 to 15 million people, HTLV-1 remains a neglected disease [177]. Similarly to HIV infection, following acute HTLV-1 acquisition, chronic infection occurs and notably, it is not a self-limited infection [178]. Most HTLV-1 positive patients may not exhibit clinical signs; however, immunosuppression or elevated viral loads resulting from immunosuppressant medications or co-infection with HIV can result in disease development [179]. Fewer than 10% of individuals infected with HTLV-1 are likely to develop two serious illnesses: adult T-cell leukemia/lymphoma (ATLL) and a subacute myelopathy referred to as tropical spastic paraparesis (TSP) or HTLV-1-associated myelopathy (HAM) [180]. HTLV-1 is efficiently transmitted sexually (more often from male to female), perinatally during breastfeeding, through transfusions, transplants, and via injection drug use [181]. These viruses are endemic in regions like Japan, the Caribbean, Sub-Saharan Africa, and Central and South America.

#### 2.6.1. HTLV-Related Cancers in Transplantation

HTLV-1 promotes oncogenesis through the Tax and HTLV-1 basic leucine zipper factor (HBZ) proteins, which dysregulate the NF-κB, cAMP-response element-binding protein (CREB), and Wnt/β-catenin pathways, driving clonal proliferation of infected T-cells while suppressing antiviral immune responses [182].

T lymphomas and rapid-onset subacute myelopathy associated with HTLV-1 infection have both been reported following solid organ transplantation [183]. The clinical impact of HTLV-1 infection after organ transplantation is controversial and unclear [181,184]. At least three different scenarios have been described for HTLV-1 acquisition and disease in transplant recipients. These include baseline carriage of HTLV-1 by the recipient, infection from the organ donor, and contaminated blood transfusions during surgery [184]. Immunosuppressive therapies used to prevent organ rejection appear to play a significant role in the frequent and rapid onset of disease, especially HTLV-1-associated myelopathy (HAM) among these individuals. Remarkably, the clinical presentation appears to differ markedly depending on whether the allograft is determined from an HTLV-1-infected donor or it is the recipient who carries the infection. When the recipient is already infected, transplantation more commonly leads to ATLL, while when HTLV-1 is acquired from the donor, the recipient’s risk for HAM/TSP is more prominent. The risk of transmission of HTLV-1 through solid organ transplantation from an affirmed infected donor is obscure. There are accounts of multiple infections from a single donor. Naturally, due to the significant volume of blood and the lack of storage, transmission would be anticipated to be higher than following blood transfusion [185]. There is no information regarding HTLV-2 infection following solid organ transplantation. HTLV-2 is a closely related virus, commonly found among intravenous drug users [186]. More than 35 years after the discovery of HTLV-1, screening for the virus remains nonexistent or sporadic in most countries [187]. Most international transplant society guidelines currently do not provide any advice on HTLV-1 screening [181]. The Global Virus Network has recently called for more systematic HTLV-1 screening before solid organ transplantation worldwide [188]. The screening costs for HTLV-1 are small in comparison with the cost of post-transplant illness and/or death associated with TSP or ATLL following HTLV-1 infection [181,189].

#### 2.6.2. HTLV-Related Cancers: Diagnosis

The diagnosis is based on the detection of specific HTLV-1 antibodies in serum. There is neither a prophylactic vaccine nor effective antiviral therapy. Prevention focuses on screening, behavioral interventions, and avoiding high-risk exposures [190].

#### 2.6.3. HTLV-Related Cancers: Prognosis

The prognosis depends on both the recipient and the donor. Studies have revealed that donors living in endemic regions should be screened for HTLV-1 before transplantation. The survival rates of HTLV-1 seropositive recipients do not differ from those of HTLV-1 seronegative recipients [191,192]. Management of oncogene viruses is briefly described in Table 2.

## 3. Discussion

It is crucial to prioritize the preservation of life and the promotion of health in transplant recipients to minimize potential post-transplant complications. Therefore, it is necessary to thoroughly explore all potential post-transplant complications and identify effective solutions. Once a detailed understanding of oncoviruses and their mechanisms of cancer development under long-term immunosuppression in transplant tissue is achieved, it is imperative to implement an efficient and optimal strategy for supervision, control, care, and treatment. Transplant recipients may have certain vulnerabilities due to their modulated immune system; consequently, in the case of cancer, the disease may follow an altered progression path and require more complex management strategies. Transplanted recipients use immunosuppressive medications that can suppress the immune system and create an environment conducive to the reactivation of latent viruses, potentially leading to the development of malignancies [197,198].

For this reason, preventing the occurrence and infection of oncoviruses as much as possible requires close monitoring of viral loads and other significant cancer-related indicators, such as warning proteins and enzymes, and promoting a safe life style. Therefore, it is crucial to regularly screen for oncoviruses, especially during the pre-transplant stage, and to have a comprehensive understanding of the patient’s health profile to make informed treatment and care decisions [199].

Within the first month after transplantation, transplant recipients are at the greatest risk of healthcare-associated infectious pathogens due to the use of immunosuppressive medicines. This occurs because most of the latent viruses in the transplanted tissue may become active during this time. It is crucial to emphasize that monitoring transplanted individuals throughout the first one to 12 months is necessary to identify viruses, assess their load and potency, particularly those that can cause cancer. If viral infections are indicated, whether active or inactive, it is crucial to manage cancer-associated factors carefully. In the case of infection, it is essential to control viral activity throughout the person’s lifespan [13,170,200]. For these individuals, it is doubly crucial to routinely evaluate the viral loads and other significant cancer-related biomarkers, such as proteins and enzymes. Utilizing screening techniques, particularly during the pre-transplant phase, and closely monitoring the patient’s health status are primary tasks [201].

Prevention of infection or reactivation of infectious agents is a critical factor in achieving optimal outcomes in solid organ transplant recipients. Although Cytomegalovirus is the main agent causing transplant especially in lung transplant recipients, Epstein–Barr virus also remains a cause of up to 20% of post-transplant lymphoproliferative disorders in immunocompromised recipients. For instance, the highest risk is related to intestinal recipients (about 17%) and followed by lung (about 10%), heart (about 5%), liver (about 3%), and kidney (about 2%) in PTLD, which is associated with a high rate of mortality after transplantation [202].

It was revealed that seronegative recipients who received solid organs from seropositive donors are at a high risk of post-transplant infections. These disorders may involve graft rejection, organ-specific injury, or even lead to mortality. There are three different periods of clinical manifestation after transplantation due to viral infections. During the initial month post-transplantation, viral infections frequently originate from pre-existing conditions, surgical complications, and nosocomial transmission. The recipients are most susceptible to reactivation of latent viral agents that may be present in the transfused blood or even the transplanted organ between one- and twelve-months post-transplantation. Moreover, the extent of immunosuppression, administration of prophylactic antiviral therapy, and exposure to different viral pathogens acquired from the community may increase the risk of viral infections within a year following transplantation. Depending on the type of oncovirus, a specific monitoring and control system is implemented. While the cost of this monitoring system may be high and it places a burden on the healthcare system, it is relatively insignificant compared to the cost of cancer treatment [201]. It is important to know about oncoviruses and the mechanisms involved in the development of cancer, which can aid in the selection of antiviral drugs and even contribute to the design of more effective treatments [203].

By using microbiological screening, a few necessary pieces of information are developed according to prevention strategies after transplantation. There are several serologic tests for both recipient and donor before transplantation including an EBV antibody panel (EBV viral capsid antigen, ±early antigen and nuclear antigen–antibody levels), HBV antibodies (HBsAb and HBcAb), and HBV surface antigen (HBsAg), HCV antibody, CMV antibody, HIV and HTLV antibodies. However, nucleic acid testing (NAT), which assesses viral nucleic acids to distinguish viral infections from bacterial infections and can confirm the viremia status, is necessary. Furthermore, the pre-transplantation serological tests are not reliable except for HPV DNA detection [204,205,206,207].

For EBV, other risk factors are considered, such as; the type of solid organ transplantation and the duration of post-transplant immunosuppression, particularly in seronegative recipients with a seropositive donor. It is established that seronegative recipients should be routinely screened using whole blood or plasma. If EBV becomes reactivated as PTLD in transplanted recipients, combination therapy should be used, including chemotherapy, radiotherapy, and immunosuppressive agents [50,208,209].

Chronic viral hepatitis-induced liver disease is a major indicator for liver transplantation, and thus infection with HCV and HBV is more frequent among organ transplant recipients. The link between HCV and HBV and post-transplantation lymphoproliferative disorder suggests that immunosuppression is essential for the expansion of virus-induced lymphoproliferation [210,211]. Moreover, a recent case series of PTLD suggested that HCV infection dynamics may act as a cofactor for EBV as well, and require further investigation [212].

The geographic variation in post-transplant virus-associated cancers reflects complex interactions between regional viral epidemiology, host immune status, and local environmental cofactors. For instance, KS demonstrates striking geographic disparities, with high incidence rates among transplant recipients from the Mediterranean and sub-Saharan Africa compared to North America [213]. Similarly, EBV-associated PTLD shows a higher incidence in pediatric transplant populations from developing countries, where primary EBV infection often occurs post-transplantation due to a later age of exposure [214].

The burden of HBV- and HCV-related hepatocellular carcinoma in transplant recipients closely follows regional viral prevalence, with these viruses accounting for >60% of cases in Asia and Africa versus <20% in Western countries [215,216]. Furthermore, UV radiation in equatorial regions may reactivate latent Merkel cell polyomavirus through localized immunosuppression [217]. These geographic patterns highlight the need for region-specific risk stratification to establish an enhanced viral surveillance system.

Emerging evidence indicates that several oncogenic viruses associated with post-transplant malignancies can promote cancer stem cell (CSC) development, which causes tumor progression, and therapeutic resistance. The immunosuppressed environment in transplant recipients particularly favors CSC expansion, as impaired immune surveillance fails to eliminate these tumor-initiating cells, while calcineurin inhibitors may inadvertently promote CSC survival through TGF-β pathway activation [218,219]. These virus-induced CSCs exhibit enhanced DNA repair capacity and drug efflux mechanisms, which explains the frequent treatment failures and high recurrence rates of virus-associated malignancies in this patient population. Understanding these mechanisms provides opportunities for targeted therapies against viral CSC markers and their associated signaling pathways [220,221].

For instance, EBV infection has been shown to induce stem-like properties in B-cells through latent membrane protein LMP-1-mediated activation of Wnt/β-catenin and Notch signaling pathways, creating self-renewing populations that seed post-transplant lymphoproliferative disorders (PTLDs) [222]. Similarly, high-risk HPV E6 and E7 oncoproteins enhance the CSC phenotype in squamous cell carcinomas by upregulating SOX2 and OCT4 stem cell transcription factors while simultaneously inhibiting cellular differentiation programs [223,224]. In Kaposi’s sarcoma, KSHV-encoded LANA protein maintains stem-like characteristics in endothelial progenitor cells through stabilization of HIF-1α and VEGF autocrine signaling [225].

This triangle of viral persistence, immune dysfunction, and genomic instability explains the accelerated oncogenesis observed in transplant recipients for example, viral oncoproteins (e.g., EBV LMP-1, HPV E6/E7) drive genomic instability through interference with DNA repair mechanisms and inactivation of tumor suppressors like p53 and Rb [151]. Where virus-induced chronic inflammation (mediated by NF-κB and STAT3 signaling) promotes the accumulation of somatic mutations and fosters an immunosuppressive tumor microenvironment rich in regulatory T-cells and myeloid-derived suppressor cells [12].

In summary, understanding the intricate relationships between oncoviruses, host immune systems, and cancer progression is vital for creating targeted interventions that may decrease the incidence of virus-related cancers among high-risk groups, including transplant recipients [226]. Based on post-transplant infectious disease guidelines, vaccination should be withheld from recipients of solid organs for at least 2 months during the period of intensified immunosuppression [227]. Identifying the concerns of transplant recipients to inform macro-level health policies and guide health management decisions can significantly reduce financial burdens for the health system and society, as well as improve the mental health of those involved in transplantation.

In conclusion, the field of transplantation has significant gaps in terms of thorough investigation, leading to the neglect of the direct and indirect carcinogenic effects of these viruses on both transplanted and non-transplanted tissue cells. We believe there is a possibility of neglected emergent viruses that can cause cancer in the immunocompromised individual, especially through transplantation. Nowadays, there are more questions than answers regarding this issue.

## Figures and Tables

**Table 1:** Oncogenic Virus.

Virus	Family	Genome	Genome Size (Approximately)	Transmission	Incidence	Ref.
**EBV**	*Herpesviridae*	ds-DNA	172 kb	Saliva, close contact	Common/High, in solid organ	[19,20]
**KSHV**	*Herpesviridae*	ds-DNA	165–170 kb	Saliva, sexual contact, organ transplantation	Increased risk in transplant recipients	[21]
**HPV**	*Papillomaviridae*	Circular ds-DNA	8 kb	Sexual & skin-to-skin contact	Common/Increased risk in transplant recipients	[22]
**HBV**	*Hepadnaviridae*	Partially ds-DNA	3.2 kb	Blood, sexual contact, vertical transmission	Varies by region/especially in liver transplant recipients	[23]
**HCV**	*Flaviviridae*	ss-RNA	9.6 kb	Blood, sexual contact, vertical transmission	Varies by region/especially in liver transplant recipients	[24]
**Merkel cell polyomavirus**	*Polyomaviridae*	Circular ds-DNA	5.4 kb	Likely through respiratory secretions	Relatively rare/	[25]
**BK virus**	*Polyomaviridae*	Circular ds-DNA	5.2 kb	Primarily through respiratory secretions and urine	Common, individuals with evidence of past infection/1–10% of kidney transplant recipients	[26]
**JC virus**	*Polyomaviridae*	Circular ds-DNA	5.13 kb	respiratory secretions and urine	Reactivation occurs in immunocompromised patients	[27]
**HPyV6, HPyV7, TSPyV**	*Polyomaviridae*	Circular ds-DNA	5.4 kb	Likely respiratory secretions or direct contact with infected individuals	Rare/specific rates are not provided	[28,29]
**HTLV-1**	*Retroviridae*	Two copies of ss-RNA	9 kb	breastfeeding, sexual contact, and blood transfusion	Endemic in certain regions, Japan, Caribbean, and parts of Africa and South America/Rare	[30]

**Table 2:** Management of Oncogenic Viruses.

Oncogene Virus						
HBV/HCV	HTLV-I	PyVs	KSHV	HPV	EBV	
Yes	Yes	No *	No *	No *	Yes	Screen before Transplantation
Serological	Serological	Serological	Serological	Cytology	Serological	Screen Method
HBsAg, HBsAb, HBcAb IgM HBcAb IgG HCV Antibody	Antibodies/NAT	Viremia	Latent Ag/Antibodies	CIN AIN	VCA IgG VCA IgM EA IgG EBNA-1 IgG	Diagnostic Test
NAT	Not done	Not done	Not done	Skin Examination ^1^	Quantification PCR (DNA in PBMC) ^2^	Monitoring after Transplant
Yes/No	No	No	No	Yes	No	Control via Vaccine
Antiviral	No cure; antiretroviral can manage symptoms	No specific antiviral	Antiviral	No cure	Antiviral	Treatment
[193,194]	[188,189]	[195,196]	[66,73]	[22,89]	[34,36]	References

* Optional screening measures. ^1^. Viral nucleic acid testing every month until 6 months post-transplant, and every 6–12 months thereafter. ^2^. Monthly self-skin examinations, but a total body skin examination every 12 months by an expert physician. NAT: Nucleic Acid Testing, CIN: Cervical Intraepithelial Neoplasia, AIN: Anal Intraepithelial Neoplasia.

## Data Availability

Not applicable.

## Abbreviations

The following abbreviations are used in this manuscript:

GVHD	Graft-versus-host disease
EBV	Epstein–Barr virus
HHV-8	Human herpesvirus 8
HPV	Human papillomavirus
IM	Infectious mononucleosis
PTLD	Post-transplant lymphoproliferative disease
KSHV	Kaposi’s sarcoma-associated herpesvirus
KICS	KSHV inflammatory cytokine syndrome
LANA	Latency-associated nuclear antigen
ART	Antiretroviral therapy
KS	Kaposi’s sarcoma
HBV	Hepatitis B virus
HCV	Hepatitis C virus
MCPyV	Merkel cell polyomavirus
HpyV	Human polyomavirus
HTLV-1	Human T-lymphotropic virus 1
PT-KS	Post-transplant Kaposi’s sarcoma
PEL	Primary effusion lymphoma
CIM	Cell-mediated immunity
VLP	Virus-like particles
HCC	Hepatocellular carcinoma
DAAs	Direct-acting antivirals
HBeAg	HBV extracellular antigen
PyVs	Polyomaviridae family
NAT	Nucleic acid testing
BKPyV	BK polyomavirus
JCPyV	John Cunningham polyomavirus
TSPyV	Trichodysplasia spinulosa polyomavirus
MCC	Merkel cell carcinomas
TS	Trichodysplasia spinulosa
ATLL	Acute adult T-cell leukemia/lymphoma
TSP	Subacute myelopathy referred to as tropical spastic paraparesis
LMP-1	Latent Membrane Protein-1
HBZ	HTLV-1 bZIP factor
CREB	cAMP-response element binding protein
CSC	Cancer Stem Cell
CTLs	Cytotoxic T Lymphocytes
NK	Natural Killer
